# Optimization of FDG-PET/CT imaging protocol for evaluation of patients with primary and metastatic liver disease

**DOI:** 10.1186/1477-7800-4-17

**Published:** 2007-07-10

**Authors:** Russ A Kuker, Geraldine Mesoloras, Seza A Gulec

**Affiliations:** 1 Department of Radiology, Jackson Memorial Hospital, Miami, FL, USA; 2 Department of Surgical Oncology, Goshen Cancer Institute, Goshen, IN, USA

## Abstract

**Background:**

Accurate determination of the extrahepatic extent and intrahepatic distribution of disease is very important in patients with primary and metastatic liver disease for deciding whether a patient receives potentially curable surgery or palliative treatment. Our objective was to evaluate the efficacy of delayed phase FDG-PET/CT imaging in lesion detection and to define its clinical impact compared to triple-phase contrast enhanced CT (CECT).

**Methods:**

30 patients underwent delayed phase FDG-PET/CT imaging (90 min whole body scan followed by a delayed abdominal scan at 120 min). Maximum standard uptake values (SUVs) and SUV ratios between tumor and normal liver parenchyma (T/N) were evaluated. In addition, comparison was made to CECT obtained within 10 days of the FDG-PET/CT to evaluate for lesion concordance within individual liver segments (Couinaud designation).

**Results:**

Sites of primary malignancies included: colorectal (19), breast (3), pancreas (2), lung (2), carcinoid (2), cholangiocarcinoma (1), and hepatocellular carcinoma (1). There was a significant increase in SUV value of liver lesions between early and delayed acquisition (P < 0.001). Although there was not a significant reduction in liver background activity between the two studies, there was a strong increase in T/N ratio (P < 0.001) allowing better lesion detection by visual inspection. New lesions were identified in 5 of the 30 patients, which were not appreciated on the early scan. Delayed phase FDG-PET/CT identified one lesion which was not present on the corresponding CECT. Delayed phase FDG-PET/CT revealed extrahepatic sites of metastases not appreciated on CECT in 6 patients.

**Conclusion:**

Delayed phase FDG-PET/CT protocol improved lesion detectability in primary and metastatic liver disease, revealing new lesions in 17% of the patients. Moreover, FDG-PET/CT identified extrahepatic disease not seen on CECT in 20% of the patients.

## Background

The liver is a frequent site of hematogenous metastases because of its rich dual blood supply. Local endocrine factors that promote cell growth and fenestrations in the sinusoidal endothelium allow tumor emboli arriving via the blood stream to implant and multiply within the space of Disse [1]. Common gastrointestinal (GI) malignancies that metastasize to the liver include colonic, pancreatic, gastric, gallbladder, and neuroendocrine tumors. Previously, patients who presented with liver metastases were classified as stage IV disease and treatment was met with great skepticism. However, advances in surgical and medical therapies over the past two decades have provided effective treatment options. Improvements in surgical technique combined with a better understanding of intrahepatic anatomy have allowed hepatic resections to be performed with acceptable morbidity. Major hepatobiliary centers routinely report less than 5% perioperative mortality for non-cirrhotic patients undergoing partial hepatectomy [2].

Anatomic resection of liver metastases is now commonly performed in colorectal cancer patients with isolated intrahepatic disease, and the impact has been dramatic with five year survival rates up to 40% [3]. Patients with extrahepatic metastases that are amenable to resection may also benefit from partial hepatectomy and can achieve five year survival rates of 28% as reported by Elias et al [4]. Similar results have been demonstrated in resection of primary liver tumors. Tanaka et al reports three year survival rates of 89% after partial hepatectomy for hepatocellular carcinoma meeting Milan criteria (solitary tumor < 5 cm or up to three nodules < 3 cm) [5]. Surgical resection of large hepatocellular carcinomas > 10 cm have less impressive outcomes but still can achieve five year survival rates up to 28% [6].

Radiologic staging of patients with primary or metastatic liver disease is vital to determine the suitability of partial hepatectomy [7-9]. The goals of imaging are twofold: define the extrahepatic extent and intrahepatic distribution of disease. Triple-phase contrast enhanced CT (CECT) has been the mainstay of preoperative planning by providing key anatomical information. MRI can provide complementary data by evaluating the signal and enhancement characteristics of liver lesions and may better delineate involvement/invasion into adjacent vascular or biliary structures. MRI, however, has low sensitivity for detecting extrahepatic disease.

PET/CT with ^18^F-FDG is an integral tool for the staging of many malignancies. PET/CT has been shown to improve the therapeutic management of patients with colorectal cancer by detecting unsuspected extrahepatic metastases [10-16]. The value of PET/CT has been questioned for detection of intrahepatic disease because of the high background activity in the liver parenchyma due to high glucose metabolism and abundant expression of Glut-1 and hexokinase II (HK-II). To circumvent the problems inherent to detection of intrahepatic lesions on PET/CT, several authors have proposed delayed PET/CT imaging and the results have been encouraging [17-21]. The premise of dual phase PET/CT is that malignant cells should preferentially accumulate activity more than normal hepatocytes thereby improving tumor to background ratios over time. Our goal was to determine whether dual phase acquisition PET/CT can improve lesion detectability in primary and metastatic liver disease and to define its clinical impact compared to CECT.

## Methods

### Patients

149 consecutive cancer patients evaluated at the Goshen Cancer Institute over a six month period underwent dual phase ^18^F-FDG PET/CT imaging. The studied patients had known primary malignancies either hepatic in origin or extrahepatic with suspected liver metastases.

### Image Acquisition

Patients were asked to fast for a minimum of six hours prior to the study and blood glucose levels had to be less than 200 mg/dl prior to injection of ^18^F-FDG. All images were acquired using a dedicated GE Discovery PET/CT scanner. Initial "early" whole body imaging commenced 90 ± 15 minutes after injection of 15 mCi of ^18^F-FDG. An additional "delayed" scan focusing on the liver was obtained 120 ± 16 minutes after the injection. PET images were reconstructed using CT attenuation correction, dead time correction, and decay correction to the beginning of each scan.

### Data Analysis

Early and delayed images were interpreted on the GE workstation in the axial, coronal, and sagittal planes along with maximum intensity projection images. Each scan was reviewed for the presence of liver lesions. Maximum standardized uptake values (SUVs) were obtained by drawing three dimensional regions of interest (ROIs) around each lesion on the early study and the corresponding lesion on the delayed study. Lesions which demonstrated SUVs greater than background activity with a minimum value of 3 were defined as positive for metastasis. ROIs were also placed over uninvolved regions of the liver to obtain SUVs of the background normal liver parenchyma. The tumor to normal parenchyma ratio (T/N ratio) was then calculated for each lesion identified on the early and delayed studies using the following formulas:

T/N *early *= SUV tumor *early */SUV background *early*

T/N *delayed *= SUV tumor *delayed */SUV background *delayed*

Paired T-Test was used for statistical comparison of early and delayed tumor SUV values, background SUV values, and T/N ratios. P values less than 0.05 were considered statistically significant for all analyses.

In addition to ROI analysis, comparison was made to CECT obtained within 10 days of the PET/CT to evaluate for the following parameters: lesion concordance within individual liver segments (Couinaud designation) and extrahepatic sites of metastases.

## Results

30 of the 149 patients demonstrated liver lesions (13 males and 17 females, mean age 61.1 years, age range 42–86 years). Sites of primary malignancies included: colorectal (n = 19), breast (n = 3), pancreas (n = 2), lung (n = 2), carcinoid (n = 2), cholangiocarcinoma (n = 1), and hepatocellular carcinoma (n = 1).

**Figure 1:**
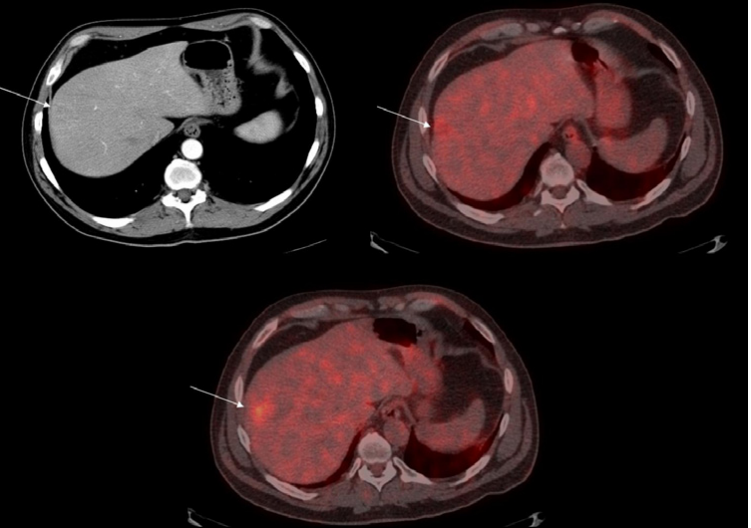
CECT (top left), early(top right) and delayed phase (bottom) FDG-PET/CT fusion images of a 46 year old male with lung carcinoma showing delayed accumulation of FDG in a segment 7 metastatic lesion.

54 liver lesions were detected among the 30 patients in our study. Five of these lesions were not apparent on the early scan and visualized only on delayed imaging (Figure 1). 46 of the 54 lesions (85%) demonstrated an increase in SUV value on delayed imaging. Further analysis of the 8 lesions whose SUV did not increase over time reveals that only one lesion was associated with an SUV decline of greater than 1 (range 0.1–2.1, median 0.3); and all of the SUV values remained above 3. T/N ratios increased in 5 of these 8 lesions allowing for better delineation on delayed imaging. The remaining 3 lesions were clearly positive on the early scan. Overall, 48 of the 54 liver lesions (89%) showed an increase in T/N ratio on the delayed phase. Background SUV values were more variable but tended to decrease in two thirds of the patients.

Table 1 shows the mean and standard deviations of maximum SUVs in malignant liver lesions and normal liver parenchyma as well as T/N ratios in both early and delayed acquisitions. Mean SUV values of malignant lesions increased from 10.5 to 11.5 on delayed imaging and demonstrated statistical significance with a P value of less than 0.001. Mean liver background SUVs decreased slightly from 3.8 to 3.6, but it did not show a significant difference (P = 0.05). However, there was a strong increase in T/N ratio (P < 0.001) allowing for better lesion detection by visual inspection on the delayed scan.

**Table 1:** Semi quantitative analysis of ^18^F-FDG uptake in metastatic liver lesions on delayed phase PET/CT.

	**Early**	**Delayed**
SUV in malignant lesions	10.2 ± 4.8	11.3 ± 5.0
SUV in normal parenchyma	3.7 ± 0.8	3.4 ± 0.8
T/N ratio	2.9 ± 1.5	3.4 ± 1.5

**Figure 2:**
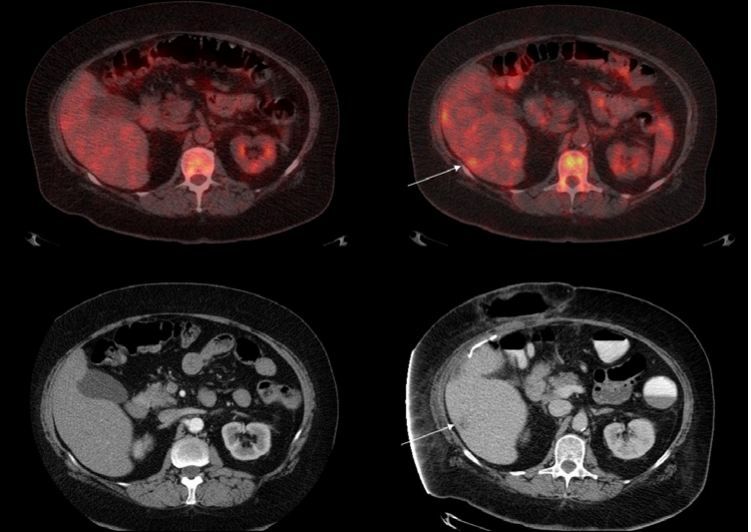
Early (top left) and delayed phase (top right) FDG-PET/CT of a 54 year old female with metastatic colorectal carcinoma showing a segment 6 lesion not seen on CECT performed on the same date (bottom left) but surfacing 6 months later (bottom right).

**Figure 3:**
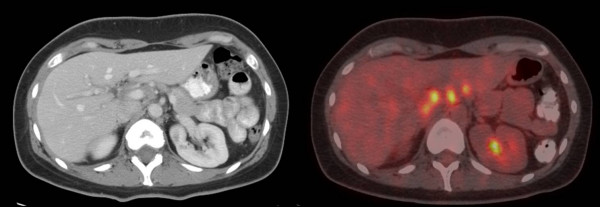
CECT (left) and delayed phase FDG-PET/CT fusion images (right) of a 42 year old female with lung carcinoma showing FDG uptake in periportal/peripancreatic lymph nodes not clearly demonstrated on CECT.

There was concordance of involved liver segments between CECT and delayed phase FDG-PET/CT in 28 of 30 patients. CECT identified more extensive disease in one patient with HCC. Delayed phase FDG-PET/CT demonstrated involvement of a new Couinaud segment in one patient with colorectal carcinoma, which was not present on the corresponding CECT performed on the same date (Figure 2). This lesion surfaced on CECT performed 6 months later confirming the validity of the PET/CT findings. Delayed phase FDG-PET/CT revealed extrahepatic sites of metastases not appreciated on CECT in 6 patients (Figure 3). Sites of extrahepatic disease not recognized on CECT included periportal/peripancreatic lymphadenopathy (n = 1), pulmonary nodules (n = 1), and osseous metastases (n = 4).

## Discussion

^18^F-FDG PET/CT is a valuable tool for the staging of many malignancies. Although the fusion of anatomic CT data with the functional information provided by PET has improved diagnostic accuracy, detection of primary and metastatic liver lesions remains challenging because of the high background FDG activity in normal liver parenchyma and reconstruction artifacts generated by respiratory diaphragmatic motion. FDG uptake in normal and malignant tissues is dependent on a variety of factors. On a macroscopic level, malignant cells metabolize glucose at increased levels because of high energy demands and therefore show PET positivity since FDG is used as an energy substrate. More aggressive tumors have higher energy demands and higher metabolic rates and tend to be strongly positive on PET. On a molecular level, it is the expression of Glut 1 and HK-II that allows FDG to enter the cell, become phosphorylated, and then trapped intracellularly allowing for coincidence detection of positron emissions. It has been suggested that Glut-1 and HK-II expression are inversely related in some primary liver tumors [22]. Cells that have high levels of Glut-1 such as mass-forming cholangiocarcinoma can easily facilitate glucose transport into the cell. High intracellular levels of glucose-6-phosphate may cause downregulation of HK-II by feedback inhibition. On the other hand, HK-II expression in high grade hepatocellular carcinoma (HCC) is elevated likely a result of upregulation caused by low levels of Glut-1 on the cell surface. Both cholangiocarcinoma and high grade HCC are strongly PET positive; and although they may have different mechanisms of FDG uptake, they have a final common pathway of increased glucose metabolism.

Is there a molecular explanation of why delayed imaging may facilitate tumor detection and why SUV values increase over time in malignant cells? The answer may lie in tumor vascularity. It has been demonstrated that Glut-1 and HK-II expression is greatest in the central region of tumors by autoradiography [23]. Aggressive tumors often have insufficient blood supply leading to hypoxia and eventual central necrosis. When tumor cells are exposed to a hypoxic environment, HIF-1α is activated to promote the transcription of glucose transporters and glycolytic enzymes. Delayed imaging allows more time for FDG to migrate to hypoxic areas which have higher regional levels of Glut-1 and HK-II. Delayed imaging also allows more time for FDG to reach hypovascular tumors or those with altered blood supply as a sequella of prior treatments. Finally, delayed imaging allows for further clearance of blood pool activity.

Dual phase ^18^F-FDG PET has been proposed by other authors for the evaluation of GI malignancies and the results have been encouraging. Nishiyama et al. reports increased lesion uptake and increased lesion-to-background contrast in gallbladder carcinoma on delayed images; however, the diagnostic performance was dependent on C-reactive protein levels [17]. Nishiyama also reports that delayed FDG PET is helpful in pancreatic cancer by identifying new metastatic foci in 3 of 55 patients [18]. In a study of 12 patients with hepatocellular carcinoma, Lin et al. found that the mean SUV, T/N ratio, and diagnostic sensitivity all increased on 2 hour delayed images. Lin et al. also showed a slight decrease in the mean SUV of normal liver tissue [19]. Our study did not show a significant difference between early and delayed background liver activity; however, this variability in background activity had little impact on T/N ratios which still increased in 86% of metastatic lesions our study. This suggests that it is the retention of FDG in malignant cells (as determined by Glut-1 and HK-II expression) rather than clearance of blood pool activity that is the most important factor in delayed lesion detection. This observation, however, needs to be correlated with biochemical analysis.

If dual phase PET/CT can improve the detection of primary and metastatic liver lesions as we have demonstrated, should all patients requiring PET/CT for staging purposes undergo dual phase acquisition? In an era where curative liver resections are increasingly performed, appropriate selection of patients for this clinical objective is imperative. The most important information provided by radiologic studies, obviously, is to determine whether an R0 curative resection can be safely performed by identifying the number and distribution of lesions and their proximity to major vascular structures and the biliary tree. In this respect, accurate staging of patients with identification of hepatic and extrahepatic distribution of lesions is very important. The ultimate answer to this question could require prospective studies with larger patient populations addressing comprehensive cost-benefit analyses. Based on the data that is available at this time, we propose that those patients who are candidates for surgical resection should undergo dual phase PET/CT with ^18^F-FDG for the following reasons. Dual phase acquisition can evaluate the metabolic activity of lesions seen on anatomical imaging and confirm benign or malignant etiology. Dual phase PET/CT may identify new metastatic lesions (intra or extrahepatic) that were not appreciated on conventional imaging modalities. In addition, dual phase PET/CT may further characterize the response of malignant lesions to neoadjuvant therapy. All of these considerations have a great impact on surgical planning and may decide whether a patient is a candidate for curative resection or whether the patient would benefit from medical therapy, decisions which are paramount for patient care and long term survival.

## Conclusion

Delayed phase FDG-PET/CT improves lesion detectability in primary and metastatic liver disease, revealing new lesions in 17% of the patients. Moreover, FDG-PET/CT identified extrahepatic sites of metastases not seen on CECT in 20% of the patients. For these reasons, we propose that patients with primary or metastatic liver disease in whom surgical resection is being contemplated should undergo staging with delayed phase FDG-PET/CT.

## Competing interests

The author(s) declare that they have no competing interests.

## Authors' contributions

RK carried out the study, performed ROI and statistical analysis, and drafted the manuscript. GM supervised and coordinated the acquisition of delayed phase FDG-PET/CT images and helped with ROI analysis. SG conceived of the study, participated in its design and coordination, and helped to draft the manuscript. All authors read and approved the final manuscript.
